# Herd and animal factors affect the variability of total and differential somatic cell count in bovine milk

**DOI:** 10.1093/jas/skac406

**Published:** 2022-12-14

**Authors:** Giorgia Stocco, Claudio Cipolat-Gotet, Bruno Stefanon, Alfonso Zecconi, Maurizio Francescutti, Maria Mountricha, Andrea Summer

**Affiliations:** Department of Veterinary Science, University of Parma, 43126 Parma, Italy; Department of Veterinary Science, University of Parma, 43126 Parma, Italy; Department of AgroFood, Environmental and Animal Science, University of Udine, 33100 Udine, Italy; Department of Biomedical, Surgical and Dental Sciences, One Health Unit, University of Milano, 20133 Milano, Italy; Associazione Allevatori del Friuli-Venezia Giulia, 33033 Codroipo (UD), Italy; Department of Veterinary Science, University of Parma, 43126 Parma, Italy; Department of Veterinary Science, University of Parma, 43126 Parma, Italy

**Keywords:** automatic milking system, breed, herd productivity, polymorphonuclear leukocyte, season

## Abstract

The aim of this study was to quantify some environmental (individual herds, herd productivity, milking system, and season) and animal factors [individual animals, breed, days in milk (DIM) and parity] on the variability of the log-10 transformation of somatic cell count (LSCC) and differential somatic cell count (DSCC) on individual bovine milk. A total of 159,360 test-day records related to milk production and composition were extracted from 12,849 Holstein-Friesian and 9,275 Simmental cows distributed across 223 herds. Herds were classified into high and low productivity, defined according to the average daily milk net energy output (D_MEO_) yielded by the cows. Data included daily milk yield (DY_M_; kg/d), milk fat, protein, lactose, SCC, and DSCC, and information on herds (i.e., productivity, milking system). The daily production of total and differential somatic cells in milk was calculated and then log-10 transformed, obtaining D_LSCC_ and D_LDSCC_, respectively. Data were analyzed using a mixed model including the effects of individual herd, animal, repeated measurements intra animal as random, and herd productivity, milking system, season, breed, DIM, parity, DIM × parity, breed × season, DIM × milking system and parity × milking system as fixed factors. Herds with a high D_MEO_ were characterized by a lower content of LSCC and DSCC, and higher D_LSCC_ and D_LDSCC_, compared to the low D_MEO_ herds. The association between milking system and somatic cell traits suggested that the use of the automatic milking systems would not allow for a rapid intervention on the cow, as evidenced by the higher content of all somatic cell traits compared to the other milking systems. Season was an important source of variation, as evidenced by high LSCC and DSCC content in milk during summer. Breed of cow had a large influence, with Holstein-Friesian having greater LSCC, DSCC, D_LSCC_, and D_LDSCC_ compared to Simmental. With regard to DIM, the variability of LSCC was mostly related to that of DSCC, showing an increase from calving to the end of lactation, and suggesting the higher occurrence of chronic mastitis in cows toward the end of lactation. All the somatic cell traits increased across number of parities, possibly because older cows may have increased susceptibility to intramammary infections.

## Teaser

The results obtained from animal-related factors stimulate the investigation of biological pathways and genetic background of total and differential somatic cell traits. Moreover, these findings address the opportunity of using the information of milk production when setting thresholds based on somatic cells for identifying udder inflammation. Hence, it could be possible to set new thresholds related to the number of somatic cells produced per day, in order to help farmers properly manage their cows and herds.

## Introduction

The advanced milk testing technology recently implemented in milk laboratories of Breeders Associations located in several countries has allowed the measurement of differential somatic cell count (**DSCC**) in milk at individual animal level (Damm et al., 2017). The rapid spread of this technology represents an important improvement in the dairy field and supports new research studies aimed at characterizing the variability of DSCC, also combined with somatic cell count (**SCC**) according to different factors (i.e., environmental, animal-related, or both factors). The variability of SCC has been described at herd (i.e., feeding, milking facility type, housing) (Alhussien and Dang, 2018a), animal nongenetic [i.e., parity, days in milk (**DIM**); Hagnestam-Nielsen et al., 2009], and genetic levels (i.e., heritability estimates, genetic correlations; Martin et al., 2018). In the last decades, the SCC has been used worldwide as indicators of udder health in dairy animals and to indirectly monitor the quality of milk, especially for cheese production (Summer et al., 2015). Also, DSCC provide more detailed insights on the inflammatory status of the mammary glands, being represented as polymorphonuclear (**PMN**) leukocytes and lymphocytes (Wall et al., 2018). Currently, thresholds based on the combination of DSCC and SCC suggested by Zecconi et al. (2019) are used within the monthly Italian milk recording system to gain more detailed information about mastitis risk assessment. Despite the growing interest in DSCC, studies about the major sources of variation of this trait at both the animal (i.e., breed, parity, DIM) and environmental (i.e., milking system, season) level are still scarce. This lack of knowledge impedes to act properly at both farm and animal level, often leading to wrong management decisions. For those reasons, a one-year study on a large number of individual bovine milk samples from Holstein-Friesian and Simmental breeds has been carried out. The specific aims of this study were: 1) to quantify the effect of individual herds and of herd-related factors, such as productivity and milking system, 2) to test the variability among seasons, and 3) to quantify the effect of the individual animal and assess associations of animal-related factors (breed, DIM, and parity) with the milk content of the logarithmic-10 transformation of SCC (**LSCC**), DSCC, and their corresponding daily somatic cell traits.

## Material and Methods

### Ethical statement

All the dairy cows involved in this study were reared in commercial private farms and were not subjected to any invasive procedures. Milk samples used for the analyses were collected during routine milking.

### Animals and herds data

Milk recording data were collected on dairy cows between July 2019 and September 2020 within Friuli Venezia Giulia, a northeastern region in Italy. A total of 159,360 test-day records related to milk production and composition were obtained from 12,849 Holstein-Friesian (**HF**) and 9,275 Simmental (**Si**) cows distributed across all the 223 herds of the region. The number of sires that had progeny with records in the data set was 1,782 for HF and 918 for Si, respectively. It is possible that the differences in the number of sires within each breed could have interfered with the breed effect. All the herds were included in the official milk test-day recording system of the region. Data and information (milking system and average herd size) on herds and cows (breed, DIM, and parity) were provided by the Breeders Association of Friuli Venezia Giulia (Codroipo, Italy). The herd milking system was classified into automatic milking system (**AMS**) (25 herds), free stall (154 herds) with milking parlor (i.e., tandem, herringbone, parallel), and tie stall (44 herds) with cow side milking (i.e., milking trolley or buckets, round-the-bar pipeline milking system). The herds were single (*N* = 103) and multibreed (*N* = 120). Herds with an average size of less than 30 cows under milk recording were not included in the study. On average, herds had about 84 cows. The average number of milk samples per cow was 7.9, with a minimum of 3 (cows with less than 3 observations were discarded from the dataset), and a maximum of 20 samples. Cows with less than 5 DIM were discarded from the analysis.

### Milk data

Milk data were provided by the Breeders Association of Friuli Venezia Giulia (Codroipo, Italy) during the routine milk recording procedures, and included daily milk yield (**DY**_**M**_; kg/d), milk composition (fat, protein, and lactose), SCC and DSCC. Milk samples were analyzed in the laboratory of the Breeders Association of Friuli Venezia Giulia. All the milk samples were collected and analyzed according to the International Committee for Animal Recording procedures (ICAR, 2020). Fat, protein, and lactose percentages were analyzed using MilkoScan FT7 (FOSS Electric A/S, Hillerød, Denmark), according to ISO 9622/IDF 141:^2013^. A Fossomatic 7DC (FOSS Electric A/S, Hillerød, Denmark; according to ISO 13366-2/IDF 148-2:^2006^) was used to measure SCC and DSCC (PMN + lymphocytes, %) and then SCC was transformed into the logarithm-10 scale [log_10_(SCC)] to LSCC. Moreover, to obtain the total and differential somatic cell produced daily, SCC was multiplied by DY_M_ while DSCC was multiplied by SCC and DY_M_. Then, they were individually transformed into the logarithm-10 scale, obtaining the **D**_**LSCC**_ and **D**_**LDSCC,**_ respectively. These traits allow to identify actual increase or decrease in total and differential cell count according to the quantity of milk produced. Computational formulas were as follows:


$$\begin{array}{l} D_{LSCC}\;=log_{10}\left(DY_{M}\times SCC\times 1,000\right) \end{array}$$

$$\begin{array}{l} D_{LDSCC}\;=log_{10}\left(DY_{M}\times \frac{DSCC}{100}\times SCC\times 1,000\right)\; \end{array}$$

For all milk traits, only data within the range of mean ± 3 SD for each log-transformed trait were considered for the statistical analysis.

### Herd productivity and season

The herds were classified into 2 levels of productivity (**HP**), according to the average daily milk energy output (**D**_**MEO**_) of the lactating cows. The net energy content (NE_L_) of milk was estimated using the equation proposed by the NRC (2001):


$$\begin{array}{ll} & NE_{L}\left(Mcal/kg\right)\;=\;0.0929\;\times \;fat,\;\%\;+\;0.0547\;\\ & \times \;protein,\;\%+\;0.0395\;\times \;lactose,\;\% \end{array}$$

Net energy (energy of 1 kg of milk) was converted to megajoules per kilogram and multiplied by the DM_Y_ of each cow (MJ/d) to obtain the D_MEO_ at individual cow level. Then, D_MEO_ data were analyzed using an ANOVA (Mixed procedure; SAS Institute Inc., Cary, NC) to obtain the least squares means (**LSM**) for all the herds after correcting for the fixed effects of season, breed, DIM, and parity, and the random effect of animal. After ranking the D_MEO_ LSM of the 223 herds, they were categorized into high (*N* = 111, average D_MEO_ = 63.19 MJ/d) or low HP (N = 112, average D_MEO_ = 34.58 MJ/d) based on the median value (40.71 MJ/d). Descriptive statistics of D_MEO_ and herd characteristics according to HP classification were presented in Table 1.

**Table 1.** Descriptive statistics of daily milk energy output (D_MEO_) and herd characteristics according to the productivity (HP) classification

	D_MEO_	
	≤40.71 MJ/d	>40.71 MJ/d
Herds, *N*	112	111
Mean	34.58	63.19
Min	20.83	40.87
Max	40.71	108.98
Breed, number of herds		
Single-breed	47	56
Multi-breed	65	55
Holstein-Friesian^1^	9	27
Simmental^1^	38	29
Milking system, number of herds		
Automatic milking system	3	22
Free stall	77	77
Tie stall	35	9

^1^The number of the herds per each breed is referred only to the single breed herds.

Seasons were defined as Winter (December to February), Spring (March to May), Summer (June to August), and Autumn (September to November).

### Statistical analysis

A univariate mixed effects model was used for the analysis of the somatic cell traits (LSCC, DSCC, D_LSCC_, and D_LDSCC_). Initially, the herd size was included in the model, but because the effect was not significant for all the tested traits, it was excluded from the statistical model. Moreover, all the ­possible interactions were tested among factors, and only those significant were kept in the statistical model, presented in the tables, and further discussed. Data were analyzed using PROC MIXED of SAS (release 9.4, SAS Institute Inc., Cary, NC), according to the following model:


$$\begin{array}{ll} & y_{mnopqrstu}=\mu +\;DIM_{m}+\;Parity_{n}+\;Breed_{o}\\ & +\;Season_{p}+\;HP_{q}+\;Milking_{r}+\;{\left(DIM\;\times \;Parity\right)}_{mn}\\ & +\;{\left(Breed\;\times \;Season\right)}_{op}+\;{\left(DIM\;\times \;Milking\right)}_{mr}\\ & +\;{\left(Parity\;\times \;Milking\right)}_{nr}+\;Herd{\left(HP_{q}\times \;Milking_{r}\right)}_{s}\\ & +\;rep{\left(animal\right)}_{t}+\;e_{mnopqrstu} \end{array}$$

where y_mnopqrstu_ is the observed trait (LSCC, DSCC, D_LSCC_, and D_LDSCC_); μ is the overall intercept of the model; DIM_m_ is the fixed effect of the mth class of days in milk (m = 1 to 12; class 1: ≤30 days; class 2: 31–60 d; class 3: 61–90 d; class 4: 91–120 d; class 5: 121–150 d; class 6: 151–180 d; class 7: 181–210 d; class 8: 211–240 d; class 9: 241–270 d; class 10: 271–300 d; class 11: 301–330 d; class 12: > 330 d); Parity_n_ is the fixed effect of the nth parity (n = 1 to 5, with class 5 including cows of parity ≥ 5); Breed_o_ is the fixed effect of the oth breed (o = HF and Si); Season_p_ is the fixed effect of the pth season of sampling (p = winter, spring, summer and autumn); HP_q_ is the fixed effect of the qth class of herd productivity level [class 1: low (≤40.71 MJ/d); class 2: high (>40.71 MJ/d)]; Milking_r_ is the fixed effect of the rth class of milking system (r = AMS, free and tie stall); (DIM × Parity)_mn_ is the fixed interaction between DIM and parity effect; (Breed × Season)_op_ is the fixed interaction between breed and season effect; (DIM × Milking)_mr_ is the fixed interaction between DIM and milking effect; (Parity × Milking)_nr_ is the fixed interaction between parity and milking effect; Herd_s_ is the random effect of the sth herd (s = 1 to 223) within the qth class of herd productivity level and the rth class of milking system; rep(animal)_t_ is the random effect of the tth repeated measurements (t= 3 to 20) within the animal; e_mnopqrstu_ is the random residual ~ N (0, *σ*^2^_e_), where *σ*^2^_e_ is the residual variance. The residual assumption checks were performed statistically. For example, the correlations between observed vs. expected residuals’ distribution under the normality assumption were 0.986 and 0.985 respectively for LSCC, DSCC, and 0.986 for both D_LSCC_ and D_LDSCC_. The covariance structure of the model was included as unstructured covariance.

## Results

### Milk composition and somatic cell traits

Descriptive statistics of DY_M_, composition, and somatic cell traits of individual milk samples are reported in Table 2. Many traits exhibited high variability (CV, %) due to the two different breeds, individual animals (i.e., protein) and farms (i.e., DY_M_). The mean value of LSCC was 4.98 (equivalent to about 95,000 cells/mL of milk) and ranged from 4.11 (5th percentile) to 6.15 (95th percentile), corresponding to SCC of about 82,000 and 1,300,000 cells/mL, respectively. The average of DSCC was 62.9%, ranging from 32.0% to 86.7%.

**Table 2.** Descriptive statistics^1^ of milk yield, composition, and somatic cell traits from individual milk samples

Item^2^	*N*	Mean	CV, %	Percentile	
				P5th	P95th
DY_M_, kg/d	159,360	20.2	55	7.60	42.5
*Milk components*					
Fat, %	159,360	3.98	19	2.79	5.28
Protein, %	159,360	3.46	11	2.86	4.14
Fat:Protein	159,360	1.16	18	0.85	1.50
Lactose, %	159,360	4.81	4	4.46	5.10
D_MEO_, MJ/d	159,360	58.0	53	22.8	118.6
*Somatic cell traits*					
LSCC, u	159,360	4.98	12	4.11	6.15
DSCC, %	159,232	62.9	27	32.0	86.7
D_LSCC_, u/d	159,360	9.22	9	8.33	10.42
D_LDSCC_, u/d	159,232	9.00	10	7.95	10.34

^1^CV, %, coefficient of variation; Percentile, 5th and 95th percentiles, which indicate the upper and lower 5% limits in the 2-tailed distribution of data.

^2^DY_M_, daily milk yield; D_MEO_, daily milk energy output; LSCC, log-10 of somatic cell count; DSCC, differential somatic cell count; D_LSCC_, daily log-10 (as unit; u) of somatic cell count; D_LDSCC_, daily log-10 (as unit; u) of differential somatic cell score.

### Effect of animal, herd, and herd productivity

The importance of individual cow on the total variability of somatic cell traits was similar among traits and ranged between 5.0% and 5.8%, whereas the variance explained by the repeated measurements was higher and ranged between 31.2% and 37.2% (Table 3). The % variance due to herd was similar to that of the animal and ranged from 5.5% to 8.2% (Table 3). The daily traits exhibited greater values because they were influenced by the high variability among herds and milk production.

**Table 3.** Analysis of variance of LSCC, DSCC, D_LSCC_, D_LDSCC_ with *F*-value and significance for fixed effects (herd productivity, milking system, season, breed, DIM, parity, DIM × parity, breed × season, DIM × milking system, parity × milking system) and the proportion of variance (in percentage)^1^ explained by herd, animal, and repeated measurements random effects

Effects	Somatic cell traits^4^			
	LSCC	DSCC	D_LSCC_	D_LDSCC_
*Fixed effects*				
Herd Productivity	12.0***	7.5***	22.8***	13.7***
Milking system	1.7	6.7***	15.6***	13.6***
Season	63.7***	1,203.1***	32.3***	133.9***
Breed	76.6***	27.6***	173.0***	148.0***
DIM^2^	648.3***	29.0***	110.0***	79.5***
Parity	583.8***	250.3***	865.6***	718.0***
DIM × parity	21.9***	3.2***	7.4***	6.6***
Breed × season	8.0***	1.3	15.0***	11.7***
DIM × milking system	0.9	3.7***	7.4***	1.3
Parity × milking system	5.1***	4.5***	4.7***	4.0***
*Random effects*				
Herd	6.5	5.5	8.2	7.9
Repeated measurements	42.2	37.0	41.9	42.2
RMSE^3^	0.43	12.9	0.42	0.49

^1^Proportion of variance for each random effect was calculated as the variance of the random effect of interest divided by the sum of all the remaining variances, including the residual.

^2^DIM, days in milk.

^3^RMSE, Root Mean Square Error. *** = *P* < 0.001.

^4^LSCC, log-10 of somatic cell count; DSCC, differential somatic cell count; D_LSCC_, daily yield of somatic cell score; D_LDSCC_, daily yield of differential somatic cell score.

**Figure 1.**
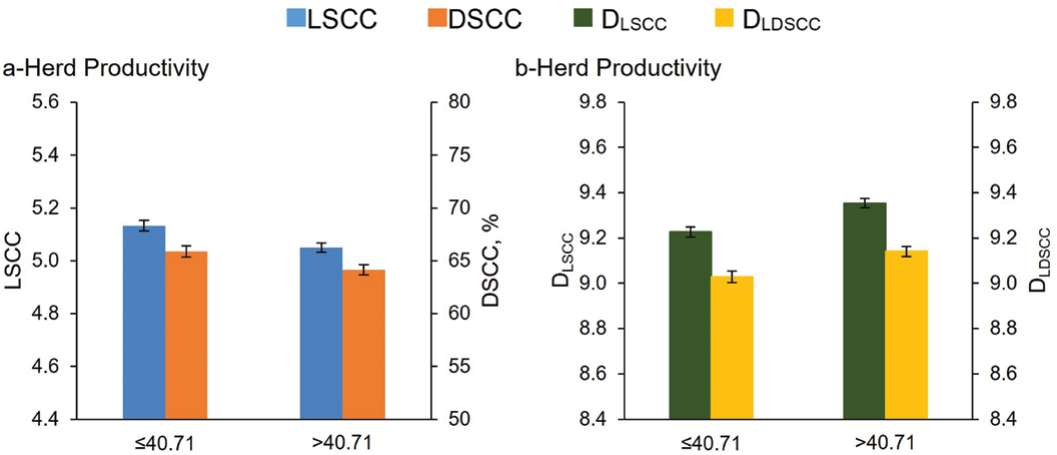
Least squares means (LSM) of LSCC, DSCC (a), D_LSCC_ and D_LDSCC_ (b) of the 159,360 milk test-day records for herd productivity (HP) level.

As depicted in Figure 1a, herds characterized by high HP level showed lower content of LSCC (5.05%) and DSCC (64.1%) compared to those belonging to low HP level (5.13% and 65.9%, respectively for LSCC and DSCC). When expressed as daily traits, D_LSCC_ and D_LDSCC_ values were higher in high-HP than in low-HP herds (Figure 1b).

### Effect of milking system and season

The type of milking system influenced the variability of all the somatic cell traits, except for LSCC (Table 3). The AMS showed higher DSCC content compared to the other two systems, while the differences between free and tie stalls were negligible for LSCC (5.08 vs. 5.04) and slightly higher for DSCC content (63.5 vs. 65.4%) (Supplemental Figure S1a). In the case of daily somatic cell traits, the differences among groups of milking system increased, with the AMS being characterized by highest content of both D_LSCC_ and D_LDSCC_, and the tie stall with the lowest (Supplemental Figure S1b).

The LSCC did not show large changes across seasons, whereas DSCC values were more variable. Indeed, DSCC reached its highest value during summer (67.8%), and the lowest value during winter (62.3%), while LSCC was slightly higher during both summer and autumn than in the other seasons (Supplemental Figure S1c). When using daily traits, the differences across seasons were more reduced, particularly for D_LSCC_ (Supplemental Figure S1d).

### Effect of breed, days in milk, and parity

Holstein-Friesian cows showed higher values for both LSCC and DSCC, and D_LSCC_ and D_LDSCC_ traits than Si (Supplemental Figures S1e and f), producing about 2.3 × 10^9^ somatic cells/d, and 1.4 × 10^9^ PMN+lymphocytes cells/d.

Days in milk were an important source of variation for milk somatic cell traits (Table 3). The LSCC decreased slightly in the first 60 DIM, moving from about 97,000 to 77,000 somatic cells/mL of milk, and then rose gradually during lactation, with the highest content toward the end (about 180,000 somatic cells/mL of milk at >330 DIM) (Supplemental Figures S1g and h). The trend of DSCC was opposite to that of LSCC, with an increase in the first 60 DIM (from 62.5% to 65.4%, for <30 to 30–60 DIM, respectively), then remaining quite stable around 65% till 300 DIM, and finally it slightly decreased thereafter.

With regard to parity, while LSCC increased linearly across parities, DSCC followed a nonlinear increase, with the lowest value for this trait in milk from second parity cows (Supplemental Figures Si and l).

## Discussion

The DSCC is complementary to SCC, and their combination provides more information on the actual immune response of the mammary gland (Damm et al., 2017; Stocco et al. 2020; Zecconi et al., 2020). It is commonly believed that high-yielding dairy cows experience more disease prevalence, shorter life expectancy, and lower environmental fitness, compared to low-yielding cows (European Commission, 2017), thus we also investigated the variability of D_LSCC_ and D_LDSCC_ traits, which are calculated from DY_M_. These traits allow the identification of an actual increase or decrease in LSCC and DSCC, according to the quantity of milk produced.

### Total somatic and differential cell count

The average DSCC value was similar to that obtained from Italian Holstein-Friesian cows reared in the Veneto region (Italy) during the routine milk test recording system (Bobbo et al., 2019). Conversely, data from routine Dairy Herd Improvement samples from France (75.0%), Denmark (72.7%), Canada (76.1%), and New Zealand (73.3%) presented higher values (Damm et al., 2017). The daily traits were 9.2 unit/d and 9.0 unit/d for D_LSCC_ and D_LDSCC_ respectively, with %CV (9% and 10%, respectively) largely lower compared to that of DY_M_ (55%). The variability of milk production was probably related to the sampling of two breeds and to the high HP differences among individual herds.

### Individual herds and herd productivity

The proportion of variance explained by herd on SCS was reported to be around 15% (Stocco et al., 2017). With regard to other milk traits, the % variance of herd usually varies between 9% and 25% for fat, protein, and lactose (Stocco et al., 2017), 25% and 75% for detailed protein profile and MUN (Amalfitano et al., 2020), 6% and 13% for coagulation properties (Ikonen et al., 2004), and 21% and 42% for cheese-making traits (Cipolat-Gotet et al., 2013). With respect of all those traits, the % variance of herd on somatic cell traits in this study was lower (Table 3), and within these traits was greater for the daily traits because they were influenced by the high variability among herds and milk production. Previous studies describing the between-herds variability for SCC (below and above 200,000 cells/mL) indicated that the farm-related factors are important for the evaluation of the dairy herd mastitis (Madouasse et al., 2010). In addition, herd milk production level was found to be a risk factor for high SCC when explaining both between-cows and between-herds performance (Madouasse et al., 2012).

The other herd-related factors tested in our study were HP level and milking system. The importance of testing the HP level relies on the fact that it greatly affects milk quality, coagulation, cheese-making, and fertility traits of cows, when herds with different milk production levels and cows with different milk yield in similar production environments are considered (Stocco et al., 2017; Stocco et al., 2018). Herds characterized by high HP level showed lower content of LSCC (5.05) and DSCC (64.1%) than low HP-level herds (5.13% and 65.9%, respectively for LSCC and DSCC; Figure 1a). The D_LSCC_ and D_LDSCC_ values were higher in high-HP than in low HP level herds (Figure 1b). The higher D_LSCC_ and D_LDSCC_ values were mostly due to the higher DY_M_ (22.3 vs. 14.2 kg/d for high and low HP herds, respectively) because cows reared in high-HP level herds showed lower number of somatic cells/mL of milk than cows reared in low-HP level herds (about 112,000 vs. 136,000 cells/mL of milk in high vs. low-HP level herds, respectively). Conversely, the DSCC, being expressed as percentage, is not associated to the dilution or concentration effect. However, when the number of differential cells/mL of milk was calculated, cows reared in high-HP level herds had a lower number of PMN+lymphocytes cells/mL of milk, compared to those reared in low-HP level herds (about 72,000 and 89,400 PMN+lymphocytes cells/mL of milk in high vs. low, respectively; data not shown). These results suggest that thresholds based on somatic cells identifying inflammation should consider also milk production because cows with different DY_M_ would have different LSCC and D_LSCC_. The use of daily somatic cell traits should be useful also considering that, the increased energy cost for the production of somatic and differential cells caused by inflammation could redirect nutrients away from milk synthesis (Johnson, 1997).

In a previous survey of six breeds of cows, where the effect of the level of HP was investigated on milk composition and coagulation traits of milk, herds with high level (average D_MEO_ = 90.86 MJ/d) had higher DY_M_, and also better milk composition and processing characteristics than those with low-HP level (average D_MEO_ = 56.35 MJ/d) (Stocco et al., 2017). Those authors suggested that the herds with high average of D_MEO_ were probably the best managed, and cows in these herds are well monitored for their health and production efficiency. Results provided by the HP factor are comparable to previous research reporting that estimates of SCC from high-yielding cows were lower than those from low-yielding cows, in animals without intra-mammary infection (Schepers et al., 1997), and confirm that ignoring this dilution effect when estimating the reduction of milk yield associated with high SCC (i.e., because of intra-mammary infection) is likely to lead to an overestimation of milk yield losses (Græsbøll et al., 2016).

### Milking system

**Figure 2.**
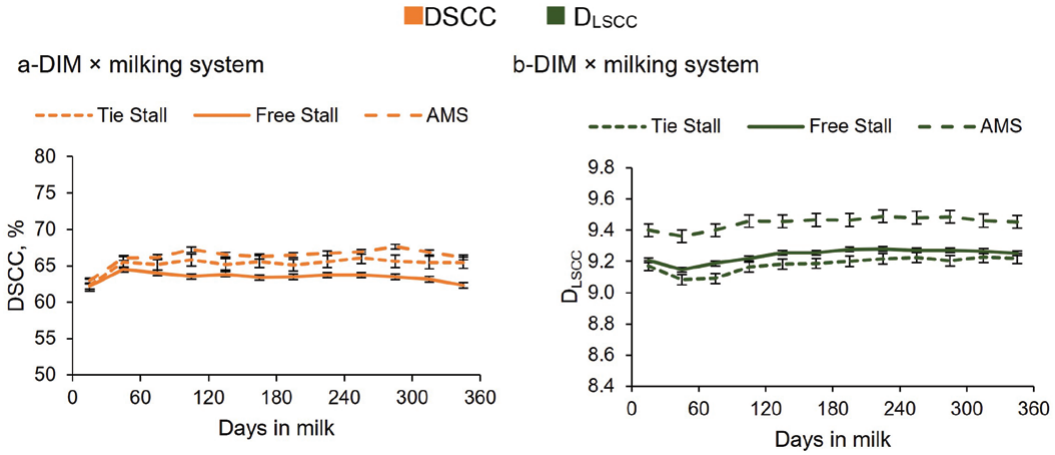
Least squares means (LSM) of DSCC (a) and D_LSCC_ (b) of the 159,360 milk test-day records for the interaction DIM × milking system.

The DIM × milking system interaction showed different trends for DSCC and D_LSCC_ and types of milking system (Table 3). In particular, cows reared in free stall showed a peak in DSCC at 60 DIM, and then a decrease toward the end of lactation, while in the case of tie stall and AMS systems, DSCC remained higher respect to the first 30 DIM, and constant throughout lactation (Figure 2a). With regard to D_LSCC_, the production of somatic cells/d remained constant throughout lactation after 120 DIM in all the three milking systems, but with substantial differences among them: 1.6, 1.9, and 3.0 × 10^9^ somatic cells/d, for tie, free and AMS stalls, respectively (Figure 2b). Also, in free stall, after the initial decrease in the D_LSCC_, the number of somatic cells produced daily remained low between 30 and 90 DIM, differently from the trends observed for tie stall and AMS types.

**Figure 3.**
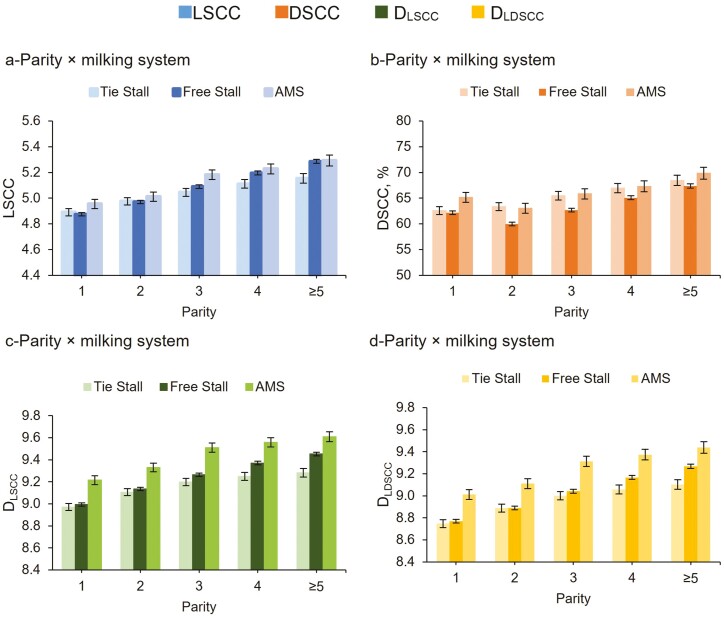
Least squares means (LSM) of LSCC (a), DSCC (b), D_LSCC_ (c), and D_LDSCC_ (d) of the 159,360 milk test-day records for the interaction parity × milking system.

The interaction parity × milking type indicated that there was a linear increase of all the somatic cell traits in the three milking type groups as the number of parities increased (Figure 3), except for the DSCC. Overall, cows reared in tie stall showed a lower content of all the somatic cell traits, and a less marked increase in their LSCC across parities (Figure 3a). This was probably due to the higher number of milkings per day, and consequently the different DY_M_ levels between AMS and free and tie stalls. The DSCC increased linearly across number of parities only within the tie stall system, whereas it was the lowest for second-parity cows (Figure 3b). Second-parity cows in free stalls showed the best health and production performance likely because of high immune system activity against stressful conditions.

### Season

**Figure 4.**
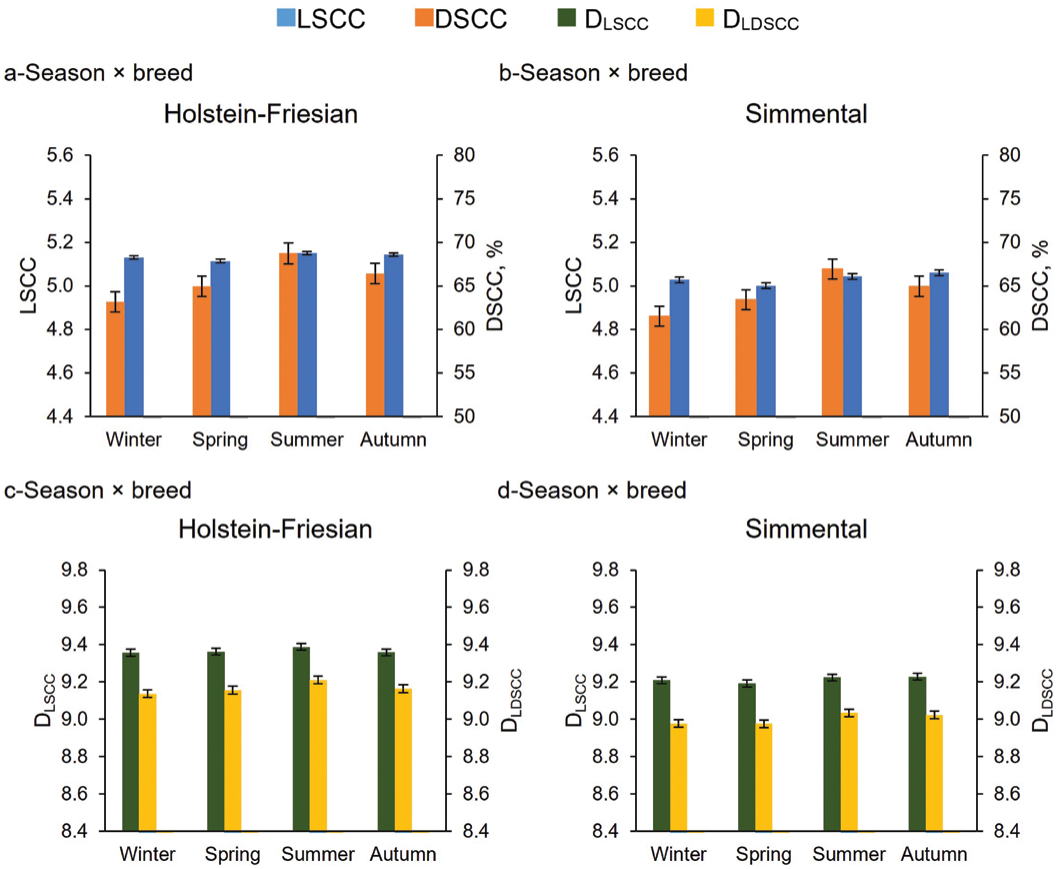
Least squares means (LSM) of LSCC, DSCC (a = HF; b = Si), D_LSCC_ and D_LDSCC_ (c = HF; d = Si) of the 159,360 milk test-day records for the interaction breed × season.

It is widely recognized that high environmental temperatures adversely affect physiological functions and productivity of dairy cows. In the present study, the interaction breed × season was significant on all the traits studied, except for DSCC (Table 3). The two breeds did show different contents of LSCC and DSCC and produced different quantities of somatic and differential cells/d across seasons, even though their trends were similar (Figures 4a and b). Also, both breeds produced the highest number of somatic and differential cells per day during summer (about 2.4 and 1.7 × 10^9^ somatic cells/d, and 1.6 and 1.1 × 10^9^ PMN+lymphocytes/d for HF and Si, respectively; Figures 4c and d), when DY_M_ was not yet at its lowest value. These results suggest that the response via cell production was similar in the two breeds, but to a different extent, due to the phenotypic and genetic differences between them (Bannerman et al., 2008). However, LSCC did not show large changes across seasons, while DSCC was more variable. The DSCC reached its highest value during summer (67.8%), and the lowest during winter (62.3%), while LSCC was slightly higher during both summer and autumn with respect to the other seasons (Supplemental Figure S1c). The high variability of DSCC across seasons was expected because this trait is not affected by the dilution or concentration respect to daily milk yield variations. Differences across seasons were more reduced for daily traits, particularly for D_LSCC_ (Supplemental Figure S1d). However, during summer, cows produced more PMN+lymphocytes cells/mL of milk (about 85,000 cells/mL) and, when expressed per day, the difference was even higher (about 2 × 10^9^ somatic cells/d and 1.3 × 10^9^ PMN+lymphocytes/d) compared to the other seasons. Hence, our results confirm that during summer (the period in which generally cows are more likely to experience heat stress), the content of cell populations in milk related to the immune response is altered. No previous studies investigated the effect of season on DSCC and daily somatic cell traits. However, although the environmental conditions were most likely not comparable to the present study, Alhussien and Dang (2018b) tested the effect of three different seasons on the same traits as in our study (i.e., SCC and DSCC), and also on cortisol and neutrophil functionality of three Indian local cow breeds, observing that all those examined traits were higher during hot dry and humid seasons compared to winter. They suggested that high temperatures, and in particular high THI (i.e., 82), led to impaired mammary defense mechanisms, which in turn increased the percentage of lymphocytes and neutrophils as well as the SCC in milk. It is important to highlight that in our study the use of LSCC was not as effective as the use of DSCC in capturing the differences across seasons. This means that the response activation from the cow is more rapid and visible through DSCC variation. Our results suggest that the possibility of gaining DSCC information, also in combination with SCC thresholds, could help in the prevention and mitigation of environmental stressors, with beneficial effects on the welfare management of dairy cows.

### Individual cow and breed

Overall, the variability of the somatic cell traits due to the animal was lower compared to herd and repeated measurements factors. In a study about the genetic variability of DSCC, Bobbo et al. (2019) found a low heritability for this trait (0.08 ± 0.02) especially when compared with milk yield and composition traits (Dadousis et al., 2018). However, our results support that a large part of the variability of DSCC trait is controlled by the animal and that further characterization and interpretations (i.e., genomic analysis) are required to better understand the relationships among the genes controlling DSCC variability. Indeed, performing a genome-wide association study on microscopic DSCC, Wagner et al. (2021) identified candidate genes for PMN and neutrophils, which are involved in pathways regulating the immune system dependent on stress level.

The literature contained no results about the effect of breed on the content of DSCC trait, but several studies can be found on the content of SCC in milk and the incidence of mastitis among different breeds. Rupp and Boichard (2003) reported that in breeds such as Montbéliarde Abondance, Si and Brown Swiss, clinical forms of mastitis are less common than in Holsteins, and their milk contains fewer SCC. Genetics could explain the differences in the content of the somatic cell traits between these two breeds. However, regardless of genetic and phenotypic differences that exist among breeds, innate immunity comprises evolutionarily primordial and preserved host defense skills (Bannerman et al., 2008).

### Days in milk and parity

The content of milk somatic cells varies during lactation and with the age of the cow, and the presence and role of each cell type within population is still under investigation under various health and disease conditions. However, to the best of our knowledge, there are no studies in the literature reporting variation of these somatic cell traits during lactation and across parities.

**Figure 5.**
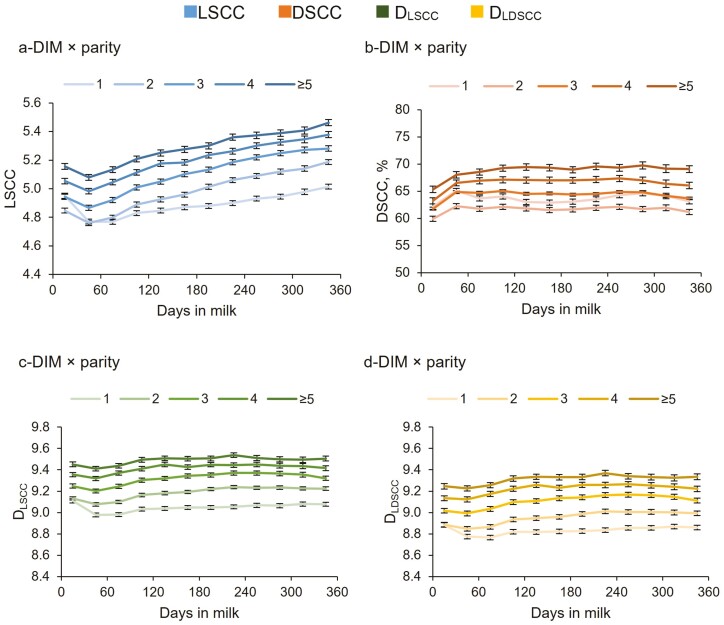
Least squares means (LSM) of LSCC (a), DSCC (b), D_LSCC_ (c), and D_LDSCC_ (d) of the 159,360 milk test-day records for the interaction DIM × parity.

The interaction DIM × parity interaction was significant for all examined traits (Table 3). There was an overall gradual increase in LSCC from the beginning to the end of lactation in different parities for all cows (Figure 5a). These findings could be explained by the higher occurrence of chronic mastitis in cows toward the end of lactation, in agreement with previous studies (Cardozo et al., 2015; Zecconi et al., 2020). Primiparous cows showed the highest decrease and the lowest increase of LSCC before and after the peak of lactation, respectively. The first calving has a high impact on the very first weeks of lactation for dairy cows, but then low values of LSCC are expected for primiparous cows because they experienced a lower number of mastitis events compared to the multiparous cows. The DSCC seemed to be quite constant throughout the lactation period (after the initial increase at 30 to 60 DIM) and showed the lowest percentage value in milk from cows at 2nd parity (Figure 5b), confirming the results for parity effect (Supplemental Figure S1i). Cows at their 1st parity had much higher DSCC compared to the secondiparous cows, and they had also higher DSCC content at the beginning of lactation compared to the tertiparous cows (Figure 5b). Probably, cows at the second parity are at the best interval time in terms of health and performance of their productive life, as the activation of the immune system is linked to an effective immune response against stress conditions. The number of somatic and differential cells increased at each additional parity (Figure 5c and d) probably because of the higher incidence of subclinical and clinical mastitis (Lee and Kim, 2006). These results agree with those reported elsewhere (Kirkeby et al., 2020; Schwarz et al., 2020), and confirm that older cows have generally a higher frequency of subclinical mastitis and are at a higher risk of becoming chronically infected compared to younger cows. This interaction also suggests that probably, the activation of the immune system in young animals at the beginning of lactation is mostly beneficial and linked to an effective immune response against stress conditions. Conversely, older cows have a higher risk of becoming infected or developing chronic mastitis towards the end of lactation. The immune dysregulation process is accelerated considerably due to long-term exposure to stressors such as previous infections, metabolic disorders, mechanical injuries, heat stress, and strain related to high productivity (Cardozo et al., 2015).

## Conclusions

Results provided by the present study showed that individual cow had greater influence than herd on the somatic cell traits examined, suggesting that a large part of the variability of LSCC and DSCC is controlled by the animal. Herd productivity levels indicated that herds with a high average of daily milk energy were probably the best managed and indicated the possibility of using the information of milk production when setting thresholds based on somatic cells identifying inflammation because cows with different DY_M_ would have different LSCC and D_LSCC_. Thus, it would be possible to set thresholds related to the number of cells produced per day. Parity × milking type and DIM × parity interactions showed the usefulness of using information provided by LSCC and DSCC in combination with daily traits during lactation, especially in older animals towards the end of lactation, and in those cases where the milking of the cow is entrusted to automation. Moreover, because the response via cell production across seasons was similar between the two breeds, it would be important to consider the differences among breeds when constructing threshold for DSCC in combination with SCC.

## Supplementary Material

skac406_suppl_Supplementary_Material

## Abbreviations

AMS: automatic milking system
DIM: days in milk
D_LDSCC_: daily logarithmic-10 transformation of differential somatic cell count
D_LSCC_: daily logarithmic-10 transformation of somatic cell count
D_MEO_: daily milk energy output
DSCC: differential somatic cell count
DY_M_: daily milk yield
HF: Holstein-Friesian
HP: herd productivity
LSCC: logarithmic-10 transformation of somatic cell count
LSM: least square means
MUN: milk urea nitrogen
PMN: polymorphonuclear
SCC: somatic cell count
Si: Simmental
