# Supplementary material for: Herd and animal factors affect the variability of total and differential somatic cell count in bovine milk
Source: J Anim Sci. 2022 Dec 14;101:skac406. doi: 10.1093/jas/skac406 (PMC9838804; doi:10.1093/jas/skac406)
Supplement: skac406_suppl_Supplementary_Material [file skac406_suppl_supplementary_material.docx]

**Supplemental Material**

**Figure S1.** Least squares means (LSM) of LSCC, DSCC, D_LSCC_ and D_LDSCC_ of the 159,360 milk test-day records for the main effects: milking system (a; b), season (c; d), breed (e; f), days in milk (g; h), and parity (i; l).

| a-Milking system | b-Milking system |
| --- | --- |
| c-Season | d-Season |
| e-Breed | f-Breed |
| g-Days in Milk | h-Days in Milk |
| i-Parity | l-Parity |
